# Using Appreciative Inquiry to Explore Effective Medical Interviews

**DOI:** 10.3390/bs11090116

**Published:** 2021-08-24

**Authors:** Masud Khawaja

**Affiliations:** University of the Fraser Valley, Abbotsford, BC V2S 7M8, Canada; masud.khawaja@ufv.ca

**Keywords:** doctor-patient relationship, social support, empathy, trust, active listening, nonverbal cues, mutual respect, confidentiality, treatment adherence

## Abstract

The objective of this study was to uncover the elements of successful medical interviews so that they can be easily shared with health educators, learners, and practitioners. The medical interview is still considered the most effective diagnostic tool available to physicians today, despite decades of rapid advancements in medical technology. When the physician-patient interaction is successful, outcomes are improved. Semi-structured interviews were conducted using an Appreciative Inquiry approach, which seeks to uncover strengths from positive experiences. The inquiry sought to identify the elements that comprise the participating physicians’ most successful patient interviews. Subsequent qualitative analysis revealed eight themes: social support, mutual respect, trust, active listening, relationships, nonverbal cues, empathy, and confidentiality. These themes do not each exist separately or in a vacuum from one another; they are in fact strongly interconnected and equally important. For instance, if a physician and a patient cannot at least maintain mutual respect, then building a relationship, or even trust, is impossible. Given the qualitative nature of this study, future quantitative research should seek to validate the results. As patients assume a more participatory role in modern medical encounters, communication and other soft skills will be key in satisfying patients and improving their medical outcomes.

## 1. Introduction

Throughout the rise of modern medicine in the nineteenth century, the medical interview has long remained a physician’s most effective tool for both rapport-building and diagnosis [1]. Even in today’s high-tech world, this human-to-human interaction remains central to therapeutic relationships. Evidence suggests that in 90% of cases, physicians reach an accurate diagnosis during a medical interview alone [2]. Thus, thorough interviews where both parties participate in the conversation, result in better health outcomes for the patient [3]. Effective conversation is not unilateral, and the forces that moved medicine away from paternalism are to thank for improved, participatory interview processes. In partnership with their physicians, patients are now playing a more active role in their diagnosis and treatment, leaving them more satisfied and with an increased likelihood of treatment adherence [4]. Given these benefits, physicians cannot let the opportunity to make partners out of patients go to waste. As the medical professional in the relationship, it is the responsibility of the physician to facilitate these collaborative outcomes [5].

Such a partnership may not occur naturally if the physician is not adept at interviewing techniques. For truly effective interviews, a strength-based approach is best utilized as it can generate positive, effective, and sustainable results [6]. Whatever diagnostic method is chosen, it should never be considered an alternative to honest and open communication. Health educators have identified physician dependence on technology as a significant impediment to effective and informative patient dialogue [7]. Should this disruption impact the diagnosis process, physician-patient relationships are sure to suffer. When patients are dissatisfied with a medical experience, they are ultimately less likely to trust their physician [8]. Degradation of this crucial relational facet is further damaged if ineffective communication is translated as a lack of transparency [9]. Thus, any barriers to participatory and transparent communication must be identified and eliminated to promote a trusting relationship that improves patient outcomes.

Since the onset of the COVID-19 pandemic, it has become increasingly apparent that mastery of clinical interview skills is needed to overcome new communicative challenges. Thus, physicians must now adapt their communicative skills to build meaningful relationships with patients even in the absence of physical meetings. With the patient in mind, this study was envisaged to determine the commonalities of the most effective medical interviews. Uncovering these elements will not only reveal barriers to effective physician-patient communication, but more importantly, indicate what is required for excellent medical encounters to occur. This qualitative study investigates effective medical interviews through the lens of physicians’ experiences, focusing on the techniques that they found most advantageous. The aim of this study is to understand and share these insights in such a way that they are easily integrated into future practice, which will ultimately improve the patient experience. 

## 2. Materials and Methods

### 2.1. Method

When evaluating the quality of medical interviews, a deficit-based approach is typically employed [10]. This approach examines poor experiences to identify the elements which lead to dissatisfaction and to generate recommendations that remedy the issues. For instance, a physician may find that an interview went poorly due to a patient refusing to discuss symptoms in favor of details that are of less diagnostic value. Based on this analysis, methods to refocus the discussion toward medically relevant details will likely be suggested [11,12]. However, this dated method of analysis ultimately robs both the participants and the analyst of rich discussion. Further, deficit-based analyses can be counterproductive: for example, students who employ a deficit-based approach, where they focus on their weaknesses and attempt to improve upon them, face worse academic outcomes than their peers who practice the opposite strategy [13]. In general, people will perform better when they seek to build upon strengths rather than just eradicate weaknesses. 

This study, therefore, employs a strength-based positive psychology approach, which focuses on the conditions that contribute to optimal results [14]. Specifically, an Appreciative Inquiry (AI) interview method was chosen, which uncovers elements that result in a positive experience, rather than focusing on what went wrong in a negative experience [15]. AI is grounded in social constructionist epistemology, which utilizes a positive perspective to understand transformational change. This method was chosen for the study because it provides the necessary foundation to explore what makes for best practices in medical interviewing, rather than taking a more traditional, negative, problem-solving approach [16]. In doing so, physicians can deeply relate to and better diagnose their patients by learning the positive elements that optimize medical interviewing skills. The emergent recommendations do not attempt to remediate failures, but rather share what works well and how to replicate positive experiences.

### 2.2. Participants

A convenience sample of six clinical faculty members was used for this study. They were generally specialists working in one of two teaching hospitals. Participants were majority male (83.33%), with an age range of 35 to 65 years (mean age: 51.67 years). Participant interviews were not anonymized or recorded, and a notetaker was present alongside the principal investigator for all interviews. 

### 2.3. Interview Process

A semi-structured interview method was used. Each physician was asked about their best interview experience, as determined by their own assessment. Where needed during the interview, follow-up questions were asked to fully understand the elements (actions by physician, actions by patients, emotions felt by physician) present and if they contributed to the outcome. They were further asked what exactly made it so successful, and how medical training can be adapted so that such interactions can become the norm. The aim of this line of probing was to elicit information about the specific elements that made those experiences so successful; this method also ensured themes could be easily identified during the subsequent thematic analysis process.

### 2.4. Thematic Analysis 

Braun and Clarke’s six steps [17] were followed to thematically analyze the interview transcripts, as recorded by the notetaker. Three researchers conducted the analysis independently and then shared the findings, which helped in triangulation of the results. The entire process involved the following phases: familiarize oneself with the data, generate code data, form themes, examine and review themes, name the themes, and uncover exemplars [17]. Researchers firstly read the transcripts several times to become familiar with the data. Then code data was generated by noting where similar discussions took place within the interviews. Code data was subsequently refined into themes. During this stage, data was mapped out and compared against the initial code data to ensure themes were consistent and unique in the dataset. The themes were then labeled, and details added for an accurate understanding of each of them.

## 3. Results

Eight themes emerged from the thematic analytical process (see Table 1). These themes provide insight into what makes an effective medical interview. They are social support, mutual respect, trust, active listening, relationships, nonverbal cues, empathy, and confidentiality. Each theme was deemed important from the participating physicians’ perspectives. 

Physicians indicated that the availability of *social support* within a patient’s network was essential for treatment adherence and success. In addition to minding physiological needs, according to a participating physician, “Family and friends should be used to augment the care that can be provided to the patient”. Physicians often probed patients about the availability of such social resources. Elderly patients represent an especially vulnerable demographic when these resources are lacking. In one case, following probing by a physician, a gravely ill elderly patient revealed that he had no remaining family except for an estranged son. In this case, the physician stated that they “decided to be proactive and instructed nursing staff to contact the son”. The outcome was not only medically beneficial for the patient, but the physician also remarked that it “brought a lasting and positive change in the patient’s life”. In a similar case, a patient facing a serious illness was not ready to accept the diagnosis and the physician felt that social support was required to move forward with the medical treatment. A patient’s family member was contacted with the patient’s consent, bridging a communicative gap that allowed for treatment to begin.

Taking the extra step to ensure patients are supported socially is only one way to show they are more than just a disease entity. This, and many other acts, display *mutual respect.* “One should not refer to the patient by his/her disease status,” said one physician. Opportunities to show patients you care for them can make a world of difference. Regarding a dying patient, conflicted about their death, one physician stated, “I lent my ears to listen to his grief. The catharsis that he had after relating his story, was something that according to him none of the other physicians had offered him”. This act of compassionate listening afforded a dying man some peace. To establish any relationship, mutual respect is of essence.

The overuse of medical terminology is counter to the idea of *mutual respect*. This sentiment was succinctly expressed by one participant during the investigation, “Healthcare professionals should not use medical jargon which patients would be unable to understand”. Patients’ level of medical understanding will vary; physicians should be attuned to determining this level and adjust accordingly. Though subtle, this type of adjustment is invaluable as the physician is able to communicate unique respect to each of their patients.

Transparency, mutual respect and *trust* were found to be inextricably linked during the semi-structured interviews. One participant described “the act of wholeheartedly welcoming the patient, handshake, frank, and open communication” as key to creating a transparent, trusting environment. This demeanour communicates many things to the patient. One physician who managed to connect with a closed-off patient remarked that “an atmosphere must be created in which the patients can speak of their deep-seated apprehensions and desires”. In line with this notion, a male physician noted that he chose to have a female nurse as a chaperone in the room after noticing a female patient’s discomfort when discussing abortion. This adjustment helped ease the patient and enhanced the communicative process. By changing the environment, the patient was more comfortable communicating openly, resulting in a smoother interview process. Speaking further about this situation, the physician mentioned that “the behavior and demeanor of the doctor is central to the development of [a] trusting relationship”.

Strong *relationships* result in better treatment adherence and patient outcomes. Once rapport is built during the medical interview, this ‘caring link’ will comfort patients as they undergo medical treatment. Again, creating an open environment is necessary for the patient to relieve themselves of “deep-seated apprehensions”. Physicians must utilize different approaches when conducting medical interviews to foster strong relationships, tailoring their methods to the patient’s particular situation. Some patients may need specific accommodations, such as one described by a physician alluding to a patient who did not fluently speak the language in which the communication was taking place, “Hand gestures and sign language can sometimes be used as an alternative if a translator is not readily available”. Whatever barriers that may exist, physicians should seek to overcome such challenges, where possible, to provide equitable medical treatment for all patients and overcome factors that inhibit relationship development.

*Active listening* is another key facet of many of the themes discussed. Without it, mutual respect is not displayed, relationships are not built, and trust will not flourish. One physician explained, “Physicians must not be preoccupied with something else or anxious when interacting with patients”. Physicians must make a patient feel heard. Moreover, they must remain present, both physically and emotionally, when communicating with patients. Active listening can be displayed in many ways. For example, a simple nod of the head demonstrates to the patient that they are understood. Poor listening habits may include focusing on a computer screen while the patient is explaining medical issues or pausing the interview to take a phone call. These behaviors tell the patient that they are not the physician’s top priority at that moment.

*Nonverbal cues* are related to the idea of active listening. The interviews uncovered the need for physicians to observe and interpret patient body language, including physical demeanor, gestures, and facial expressions. As explained by one participant, “A physician should be observant enough to get cues from the patient’s body language and eye movement”. Physicians can also use nonverbal cues to improve communication. During one interview, a participant made the following statement, “[A patient] should regard the physician as a friend and healer and not as a superior being”. Physicians can adapt their body language to indicate friendliness by facing the patient directly, sitting down instead of standing, and kneeling beside or at the eye level of young children. If the physician is frowning, the patient may assume they said something wrong. Just as the patient’s nonverbal cues influence physician perceptions, the opposite is also true; adjusting verbal tone and body language based on the patient’s reaction is a valuable skill. In short, if a patient seems uncomfortable, physicians should be cognizant of such signals and make changes. In the physician-patient relationship, the onus to adjust is on the physician as they are the skilled care-provider. Patients are not uniform but rather complex individuals requiring personalized approaches, especially if something is noticeably amiss in their behavior. Each patient brings unique needs and characteristics, and physicians must continually communicate both verbally and nonverbally with patients to adapt to those needs.

A study participant commented that “it is imperative that learning the techniques to empathize with patients be made an integral element of medical training”. *Empathy* requires that physicians relate to their patients and genuinely understand the emotions that they are feeling. In this way, patients can be better comforted, allowing for enhanced communication during medical interviews. Participants explained that a physician must demonstrate that they are always acting in the patient’s best interest. For example, one physician made the following statement, “Going the extra mile certainly paid off and helped improve the patient’s quality of life”.

In another interview, by empathizing with a terminally ill patient, the physician was able to give him the courage to face challenges that lay ahead, resulting in a positive experience amid a bleak prognosis. Patients often lack a complete understanding of a given medical process, so empathy is required to recognize if there is some confusion; the process can then be explained in a different way to help ease anxiety resulting from the confusion. Physicians can express empathy by being supportive, providing comfort and feedback, and keeping patients informed regularly. Such empathetic communication strengthens the physician-patient relationship. 

The theme of *confidentiality* also emerged in the interviews, with one participant noting that “privacy and confidentiality of the physician-patient relationship must be maintained”. Patients who do not trust their physician to maintain confidentiality may withhold crucial information, potentially affecting proper diagnosis and treatment. For example, if patients do not inform physicians about their socioeconomic conditions, then it could potentially result in treatment non-adherence if expensive medications are prescribed in the absence of insurance. During medical interviews, it may be necessary for physicians to communicate that all information will be kept private if the patient appears uneasy regarding this matter.

Assuring the patient of confidentiality may also help to minimize dishonesty. Dishonesty is a continual barrier to both effective communication and accurate diagnosis. Patients may lie for various reasons, including fear of their information being shared outside of the physician-patient relationship. For example, a teen may lie about sexual activity for fear of a parent finding out. The physician must overcome this challenge by communicating the importance of trust and confidentiality in the relationship. Honesty is an essential component of the medical relationship, and when extended it will be well reciprocated.

## 4. Discussion

All of the themes identified are interconnected with one another. Failure in one regard is doubtlessly failure in another, or many. The inability to build a relationship with a patient may stem from any number of failures related to skills pertinent to one of these themes. Therefore, equal credence should be afforded to all of them. Over the past few decades, the public’s overall trust in medical professionals has declined [18]. This signals a need to search for ways to improve patient satisfaction, as was the aim of this study.

The first theme revealed that social support is a requisite for successful treatment. This result was echoed in Turan et al.’s [19] study, which found that treatment adherence positively correlates with social support. Furthermore, it is known that patients with little or no social support are at a notable disadvantage compared to those patients that already have a strong social network [20]. The patients who experienced a lack of social support in this study were all elderly, but younger patients could experience the same void. When support is lacking, healthcare workers can act as a surrogate social supporter [21]. During the COVID-19 pandemic, the elderly demographic has been hit particularly hard in terms of isolation; this highlights the need for supportive healthcare workers, now more than ever before.

**Figure 1:**
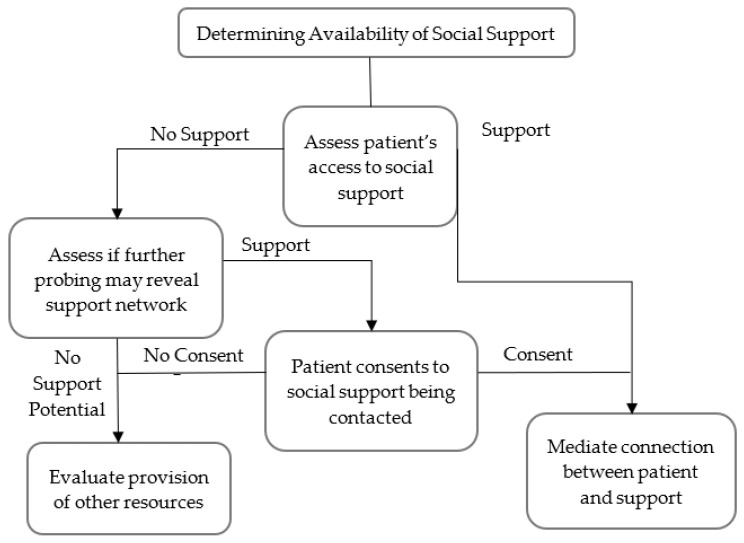
Framework flowchart for assessing patient support network.

When necessary, a physician may also assist a patient in strengthening that network if it is lacking. This was the case in two of the interviews. However, physicians must tread carefully, or they risk overstepping social norms and devastating the physician-patient relationship. In particular, family relationships can be complex, and it may prove inappropriate to contact the patient’s family in some situations. Thus, determining the availability of social support in a medical interview is a critical yet hazard-prone step. Figure 1 is based on the content of the interviews, encompasses the theme, and offers a general procedure for navigating this situation.

Trust was observed to be interconnected with other themes and is known to be foundational to successful medical interactions with patients [22]. Physicians must communicate that they are competent and well-informed to establish trust. Professionalism is one such tool to facilitate this outcome [22]. However, in displaying professionalism, communication should never be hindered. As previously stated, overreliance on medical jargon will impair the conveyance of mutual respect. Studies also support the notion that communication stemming from a trusting relationship can reduce laboratory tests required to reach a diagnosis [18]. Likewise, related to trust and addressed as a theme, confidentiality will also result in better experiences for patients [23]. Moreover, research on patient communication increasingly emphasizes adaptation based on individual needs [24]. Failing to do so could result in poor communication or understanding, with consequent worse outcomes [25]. All these factors are a lot to balance for anyone, but skillfully integrating all the themes discussed stands to improve interactions with patients.

Patient satisfaction in general is associated with better communication during medical encounters [26]. Likewise, the patient will view interactions poorly when active listening skills are not employed [27]. This sentiment was shared in the physician interviews. In the realm of listening and communication, physicians need to recognize signs of discomfort through adept empathy skills and by knowing patients well enough to see when their nonverbal behavior differs from the norm [28]. This is particularly important for vulnerable patients. For example, a person being abused by a partner or parent may exhibit different nonverbal cues depending on whether the abuser is also in the room. When the physician recognizes such cues, they can act in a trauma-informed way to preserve the physician-patient relationship [29]. Furthermore, research indicates that empathetic interactions are beneficial for all types of patients, not just those with terminal illnesses [30]. This may help build trust and create a sense of confidence.

AI was the chosen methodology for this study as its focus is to enact positive change for organizations. Working toward this change differentiates it from other methods of inquiry. Phenomenology, for instance, seeks a similar outcome of deciphering an experience, but lacks the underlying motivation to change organizations [31]. A narrative style is strong when seeking to capture many details and captivate the audience [32]. However, the experience captured with AI is not something that can be readily imparted. A measure of analysis is needed to distill experiences into their most transferable elements of knowledge, where consensus has deemed them important. The aim of this study was to uncover these elements so they may be easily shared. Thus, AI was a suitable choice to fulfill this objective.

Physicians cannot rely solely on medical knowledge or technology for successful patient interactions. Medical interviews are used not only to diagnose issues quickly but also to increase patient satisfaction. The results of this study signal a need to maintain soft skills, such as empathy, alongside medical expertise. Doing so may prove especially advantageous for assessing and supporting patients’ mental health. The COVID-19 pandemic and social distancing protocols have frayed social support networks for many. This change is contrary to one of the most prominent themes of patients’ need for social support. This is especially important for individuals who demonstrate mental health disorders and mental illness. For over a year now, isolation has become standard in our Western society, which has negatively impacted many people’s mental health. Unfortunately, as the threat of COVID-19 subsides, regaining social support may prove difficult for some, leading to sustained mental health issues. Physicians can support patients’ mental health by engaging in both social and medical topics during the medical interview, and suggesting avenues for treatment if mental health issues are present.

However, connecting with patients in this manner may come at a mental health cost for physicians themselves. Like their patients, physicians have also experienced a mental health toll during the pandemic, leading to increased stress and burnout symptoms. Workplace stress and burnout have long been attributed to high-empathy careers and tempering in this area may be required if stressors unduly burden physicians [33]. Working with many patients with serious issues can weigh heavily on a physician, especially if they take excessive personal responsibility for patients’ well-being. Thus, it is beneficial to partake in stress and burnout avoidance training programs to ensure a healthy work environment. Such training should educate physicians to take responsibility for patient outcomes without feeling unnecessarily responsible for factors beyond their control, such as terminal illnesses. The best outcomes for patients will be out of reach if burnout impairs work quality. Physicians must learn to self-assess and take individual or organizational steps to deal with symptoms of burnout, if present [34].

### Strengths, Limitations, and Future Research

This study may be limited in its generalizability for several reasons. As physicians were asked to evoke their own best experience and describe it in a conversational style, results were vulnerable to distortion due to social-desirability bias; especially since the conversation relates to the physician’s own medical interviewing prowess. The sampling method also limits this study; however, this is offset to some degree by the narrow aim of the study and by the established interview method delivered by a credentialed interviewer [35]. The topic could benefit from future quantitative or mixed-methods studies. Mental health, psychological trauma, and other ailments also represent rich quarries of analysis to determine how cognitive patterns and behaviors affect patient communication in medical settings. Larger studies regarding complex medical issues, both physical and psychological, will provide further insight into how medical interviews can be navigated in difficult cases. Barriers to face-to-face communication, such as during the COVID-19 pandemic, may also be specifically analyzed, to determine how physical boundaries have affected relationships between physicians and patients. By exploring these areas, new information can emerge, resulting in more comprehensive medical interviews and improved patient outcomes.

## 5. Conclusions

As technology pervades our lives evermore, soft skills such as communication must never degrade through neglect. At the same time, opportunities to harness technology as a communicative advantage, and not a barrier, should be carefully considered. Using technology to augment the power of an effective medical interview can assist physicians in fine-tuning their skills. The theme of nonverbal cues indicates the importance of reading and communicating body language. Facial recognition technology may help physicians identify minute, nonverbal cues that signify discomfort. Artificial intelligence may be able to score levels of empathy recorded in a conversation, as a training and evaluation tool. Further analysis will reveal new pathways to improved communication, but more research is required. The opportunities are limitless and the benefits to patients much the same. A proper balance between physician skills and technological advancements must constantly be maintained, with the latter never overtaking and causing the former to lapse. The themes outlined in this study provide a basis for future exploration on a larger scale using novel methodologies in effective interview mechanics. However, one standard method will never fit every unique patient, so a keen aptitude for adaptation should forever be honed to continuously improve therapeutic relationships.

## Figures and Tables

**Table 1:** Eight themes found in effective medical interviews.

Theme	Physician Action
Social Support	Probe patient to determine support network. Connect patient with social support when appropriate. If unavailable, provide other options.
Mutual Respect	Avoid overuse of medical jargon. Be aware of eye contact and body language. Address patient by name.
Trust	Demonstrate professionalism. Encourage open communication. Adapt environment to meet patient needs.
Relationship	Eliminate communication barriers. Be honest and encourage honesty from patient.
Active Listening	Listen carefully and repeat back to patient. Use positive body language. Minimize environmental distractions.
Nonverbal Cues	Assess patient’s cues. Pay attention for signs of abuse or trauma. Be cognizant of nonverbal cues in self.
Empathy	Display genuine empathy for patient. Self-assess for signs of burnout and excessive stress.
Confidentiality	Reassure patient of privacy. Address patient’s concerns.

## Data Availability

Restrictions apply to the availability of data. Data was obtained from physicians and are available from the author if authorized by the physicians.
